# Engineering better chimeric antigen receptor T cells

**DOI:** 10.1186/s40164-020-00190-2

**Published:** 2020-12-02

**Authors:** Hao Zhang, Pu Zhao, He Huang

**Affiliations:** 1 Department of Hematology, The Third Affiliated Hospital of Wenzhou Medical University, Wenzhou, China; 2 Bone Marrow Transplantation Center, The First Affiliated Hospital, School of Medicine, Zhejiang University, No. 79 Qingchun Road, Hangzhou, China

**Keywords:** Chimeric antigen receptor T cells, Acute lymphoblastic leukemia, Relapse, Persistence, Differentiation, Exhaustion

## Abstract

CD19-targeted CAR T cells therapy has shown remarkable efficacy in treatment of B cell malignancies. However, relapse of primary disease remains a major obstacle after CAR T cells therapy, and the majority of relapses present a tumor phenotype with retention of target antigen (antigen-positive relapse), which highly correlate with poor CAR T cells persistence. Therefore, study on factors and mechanisms that limit the in vivo persistence of CAR T cells is crucial for developing strategies to overcome these limitations. In this review, we summarize the rapidly developing knowledge regarding the factors that influence CAR T cells in vivo persistence and the underlying mechanisms. The factors involve the CAR constructs (extracellular structures, transmembrane and intracellular signaling domains, as well as the accessory structures), activation signaling (CAR signaling and TCR engagement), methods for in vitro culture (T cells collection, purification, activation, gene transduction and cells expansion), epigenetic regulations, tumor environment, CD4/CD8 subsets, CAR T cells differentiation and exhaustion. Of note, among these influence factors, CAR T cells differentiation and exhaustion are identified as the central part due to the fact that almost all factors eventually alter the state of cells differentiation and exhaustion. Moreover, we review the potential coping strategies aiming at these limitations throughout this study.

## Background

In recent years, chimeric antigen receptor T cells (CAR T) emerged as one of the most promising approach in cancer treatment [1]. The most impressive responses have been achieved in patients with B cell malignancy, especially in refractory or relapsed B acute lymphoblastic leukemia (B-ALL) treated by CAR T cells targeting CD19 with the complete remission (CR) rate reaching 90% [2–5]. However, ~ 30–50% of patients experienced leukemia relapse, the majority relapsed within 1 year after CAR T cells therapy [6], and with prolonged follow-up, the relapse rate may be much higher. Disease relapse following CAR T cell therapy can be categorized into two major patterns: target antigen loss relapse or antigen-positive relapse. The mechanisms associated with loss of CD19 after CART cells therapy include the deletion or mutation of CD19 gene in leukemic cells [7, 8], abnormal CD19 RNA splicing because of decreased expression of splicing factor SRSF3 [8], leukemia lineage transformation caused by transcription factors PAX5 and EBF1 associated reprogramming of the pre-B cells [9, 10]. The strategies for prevention or treatment of antigen loss relapse include administration of alternative antigen targeted CAR T cells, sequential infusion of different antigen targeted CAR T cells [11] and infusion of CAR T cells dual/multiple targeting different antigen [12]. Relapses of CD19-expressing leukemia in patients who achieved initial remission after CD19 targeting CART cells treatment highly correlates with poor CAR T cells persistence [2]. Therefore, study on factors and mechanisms that limit the in vivo persistence of CAR T cells is crucial for developing strategies to reduce the probability of tumor relapse and improve the long-term disease-free survival for patients who are treated with CAR T cells.

Human derived T lymphocytes engineered to express chimeric antigen receptors, which are expanded in vitro culture and then infused into patients exerting robust cytotoxicity after tumor antigen recognition and subsequent activation. Various factors in the process contribute to impact the in vivo persistence and durable antitumor effects of CAR T cells. In this review, we summarize the rapidly developing knowledge regarding the influence factors and mechanisms of poor CAR T cells persistence, and also the potential strategies to overcome these limitations.

## CAR constructs

### Extracellular structures

The extracellular structure conventionally includes single-chain variable fragment (scFv) and spacer. ScFv is the antigen-binding domain of CAR structure, which is composed of a single heavy and light chain of monoclonal antibody connected by a linker. A murine derived scFv has been reported to induce HLA-restricted T cell-mediated cellular immune response or humoral immune response [13], while humanized scFv is expected to reduce the immunogenicity of CAR, thereby avoiding immune mediated rejection and improving the persistence and therapeutic efficacy of CAR T cells [14, 15]. The different antigen-targeted scFv on the T cells membrane has different impact on CAR T cells fates. Sustained tonic CD3ζ phosphorylation in GD2 targeting CAR, which is triggered by antigen-independent clustering of CAR scFv, can induce early exhaustion of CAR T cells, and such tonic signaling is present to varying degrees in all CARs studied [16]. Moreover, the density of CAR surface expression which can be controlled by using different eukaryotic promoters has a substantial impact on antitumor efficacy and persistence of CAR T cells [17]. Low level expression of CARs driven by weaker rather than by stronger promoters results in significant higher numbers of circulating and tumor infiltrating CAR T cells in mice [18]. As is known that the affinity of CAR binding to antigen is the premise of CAR T cells function, but lower rather than higher affinity CAR-incorporated CAR T cells showed enhanced expansion, better overall and event-free survival and lower toxicity compared with FMC63 CAR T cells [19].

The extracellular spacer domain of CAR has no signaling function, but the length and composition of the extracellular spacer domain influence the spatial configurations and Fc receptor binding, and further the in vivo persistence and antitumor effects of CART cells [20, 21]. Therefore, optimizing the design of scFv and spacer can be effective approaches to improve the therapeutic efficacy of CAR T cells.

### Transmembrane and intracellular domains

The transmembrane domain which is usually derived from CD8 or CD28 connects the extracellular antigen binding domain with the intracellular signaling domain. Compared with T cells expressing CARs with CD28 hinge and transmembrane domains, T cells expressing CARs with CD8α counterparts produce lower levels of cytokines and exhibit lower levels of activation-induced cell death (AICD) [22].

The intracellular structure domain involving CD3ζ and co-stimulatory molecules is essential to full T cell activation. However, CAR incorporating all three CD3ζ immunoreceptor tyrosine-based activation motifs (ITAMs) may foster counterproductive T cell differentiation and exhaustion. CAR encoding a single ITAM balanced the replicative capacity of long-lived memory cells and the acquisition of effective antitumor function, favoring persistence of CAR T cells with enhanced therapeutic potency [23].

First-generation CAR T cells utilizing CD3ζ signaling alone showed limited efficacy in clinical trials probably owing to the AICD or the lack of long-term T cells expansion in vivo. The co-stimulatory molecules are critical for CAR T cells expansion and persistence following adoptive therapy. Second-generation CAR using combination of CD3ζ and an additional co‑stimulatory signaling domain such as CD28 or 4-1BB demonstrated remarkable efficacy with enhanced in vivo expansion and persistence [24–28]. Subsequently, CARs incorporating other co‑stimulatory signaling moieties such as CD27, ICOS and OX40 [29–31], or multiple co‑stimulatory molecules in tandem emerged and demonstrated different characteristics on their biological functions (Table 1). CD19-targeted CAR T cells incorporating the 4-1BB co-stimulatory domain show more persistent than those incorporating CD28 in clinical trials [16, 32, 33]. Compared to CAR T cells with CD28, 4-1BB is associated with more antiapoptotic proteins and reduced T cells exhaustion [16, 34]. Changing a single amino acid residue asparagine to phenylalanine in CD28 domain (CD28-YMFM) promotes durable antitumor effects with reduced CAR T cells differentiation and exhaustion as well as increased Th17 skewing [35]. Engineered CD28 lacking lck binding moiety (Delta-CD28) may also be a promising co‑stimulator for better CAR T cells persistence [36]. After antigen stimulation, CD27-bearing CAR T cells proliferate with upregulated Bcl-X_L_ expression and resisted apoptosis [29]. CAR containing inducible co-stimulator (ICOS) generates IL-17-producing effector cells with the characteristic of TH17 cells showing enhanced persistence in vivo [30, 37]. OX40 co‑stimulatory signaling effectively represses IL-10 secretion, and contributes to counteract self-repression, thus facilitates a prolonged CAR T cells response [31, 38]. Toll-like receptor 2 (TLR2)-incorporated CAR T cells demonstrate improved expansion and persistence due to the capacity of generating memory T cells, expressing pro-survival proteins and abolishing the suppression of regulatory T cells [39–41]. CD40 signaling contributes to memory formation and rescuing T cells from exhaustion [42, 43]. Combination of CD40 and TLR adaptor molecule MyD88 signaling in CAR T cells results in improved persistence and enhanced efficacy in hematological malignancy and solid tumor models [44, 45].

**Table 1:** Characteristics of different co-stimulatory molecules incorporated in CAR T cells

Co-stimulatory molecules	Characteristics	Author and year
CD28	Facilitates full and sustained T cell activation, growth and survival	Maher et al. (2002) [28]
CD28-YMFM	Reduces T cell differentiation and exhaustion with increased skewing toward Th17 cells	Guedan et al. (2020) [35]
Delta-CD28	Better persistence with higher expression of genes involving cell division, glycolysis, fatty acid oxidation, and oxidative phosphorylation	Gulati et al. (2020) [36]
4-1BB	Be associated with more anti-apoptotic proteins and reduced T cells exhaustion	Long et al. (2015) [16] Li et al. (2018) [34]
CD27	Upregulates Bcl-X_L_ protein expression and resists apoptosis	Song et al. (2012) [29]
ICOS	Presents the characteristic of TH17 cells with increased expression of IL-17A, IL-17F, and IL-22 following antigen recognition	Guedan et al. (2014) [30]
OX40	Represses IL-10 secretion and counteracts self-repression	Hombach et al. (2012) [31]
TLR2	Generates memory T cells, expresses pro-survival proteins and abolishes the suppression of regulatory T cells	Lai et al. (2018) [39]
MYD88/CD40	MYD88 is a TLR adaptor molecule. CD40 contributes to memory formation and rescuing T cells from exhaustion	Mata et al. (2017) [45] Collinson-Pautz et al. (2019) [44]

### Accessory structures

Considering the dependence on certain cytokines and interactions with endogenous immune cells, CAR T cells with accessory genetic modifications are designed to secrete cytokines or express ligands in order to benefit from autocrine or survive in the tumor microenvironment (Table 2). IL-7 is known to enhance the proliferation and survival of T cells, and co-expression of IL-7 improves NKG2D-based CAR T cells therapy on prostate cancer by enhancing the expansion, inhibiting cell apoptosis and exhaustion [46]. The strategy of co-expressing IL-7 receptor which triggers the IL-7 signaling axis but is unresponsive to extracellular cytokine increased the persistence and antitumor activity of CAR T cells [47]. CCL12 and CCL19 is chemoattractant for T cells and DCs. Combined expression of IL-7 and CCL21 significantly improved survival and infiltration of CAR T cells and DCs in tumor [48]. IL-7 and CCL19 expression in CAR T cells improves immune cell infiltration in the tumor, where both recipient conventional T cells and administered CAR T cells generated memory responses against tumors [49]. CAR T cells with transgenic expression of IL-15 and IL-21 show superior in vivo expansion, persistence and antitumor activity against hepatocellular carcinoma with a higher percentage of stem cell memory and central memory populations after manufacturing [50]. CAR encoding a truncated cytoplasmic domain from IL-2 receptor β-chain (IL-2Rβ) and a STAT3-binding tyrosine-x-x-glutamine (YXXQ) motif activates the JAK/STAT pathway and triggers gene expression profiles analogous to those triggered by IL-21, facilitating a better CAR T cells persistence [51]. Introduction of IL-12 into the tumor microenvironment holds the promise of improving CAR T cells cytotoxic function, mitigating Treg-suppression and reprogramming tumor-associated macrophages and dendritic cells [52, 53]. IL-23, consisting of IL-23α p19 and IL-12β p40 subunits, is known to promote proliferation of memory T cells, and engineered CAR T cells with expression of the p40 subunit show improved antitumor capacity with increased granzyme B and decreased PD-1 expression via autocrine IL-23 signaling [54]. CD40L/CD40 is important to T cells activation, proliferation and memory formation [42, 55]. The altered tumor phenotype with increased immunogenicity (upregulation of CD80, CD86, HLA and Fas) which is susceptible to apoptosis can be indued by CD40L expressing CAR T cells [56]. 4-1BBL armored CAR T cells exhibited balanced tumoricidal function and increased T cell persistence accompanied by elevated CD8/CD4 ratio and decreased exhaustion due to the induction of the IRF7/IFN-β pathway [57]. Accessory ICOSL resulted in enhanced CAR T cells activity via enhanced PI3K signaling pathway and upregulated expression of Bcl2 [58].

**Table 2:** The application of accessory genetic modifications in CAR T cells

Accessory molecules	Functions	Author and year
IL-7 or IL-7R	Inhibits T cells apoptosis and exhaustion	He et al. (2020) [46] Shum et al. (2017) [47]
IL-7/CCL12	Improves the survival and infiltration of CAR T cells and DCs in tumor	Luo et al. (2020) [48]
IL-7/CCL19	Improves the infiltration and anti-tumor response of both recipient conventional T cells and CAR T cells in tumor	Adachi et al. (2018) [49]
IL-15/IL-21 IL-2Rβ/YXXQ	Generates stem cell memory and central memory T cells activates the JAK-STAT pathway and triggers gene expression profiles analogous to those triggered by IL-21	Batra et al. (2020) [50] Kagoya et al. (2018) [51]
IL-12	Mitigates Treg-suppression and reprogram tumor-associated macrophages and dendritic cells	Pegram et al. (2012) [52] Koneru et al. (2015) [53]
IL-23	Increases granzyme B and decreases PD-1 expression	Ma et al. (2020) [54]
CD40L	Facilitates T cells memory formation and induces increased immunogenicity on tumor cells	Curran et al. (2015) [56]
4-1BBL	Increases CD8/CD4 ratio and decreases T cells exhaustion due to the activation of IRF7/IFN-β pathway	Zhao et al. (2015) [57]
ICOSL	Enhances PI3K signaling pathway and upregulates the expression of Bcl2	Hu et al. (2019) [58]

## In vitro cell culture

**Fig. 1:**
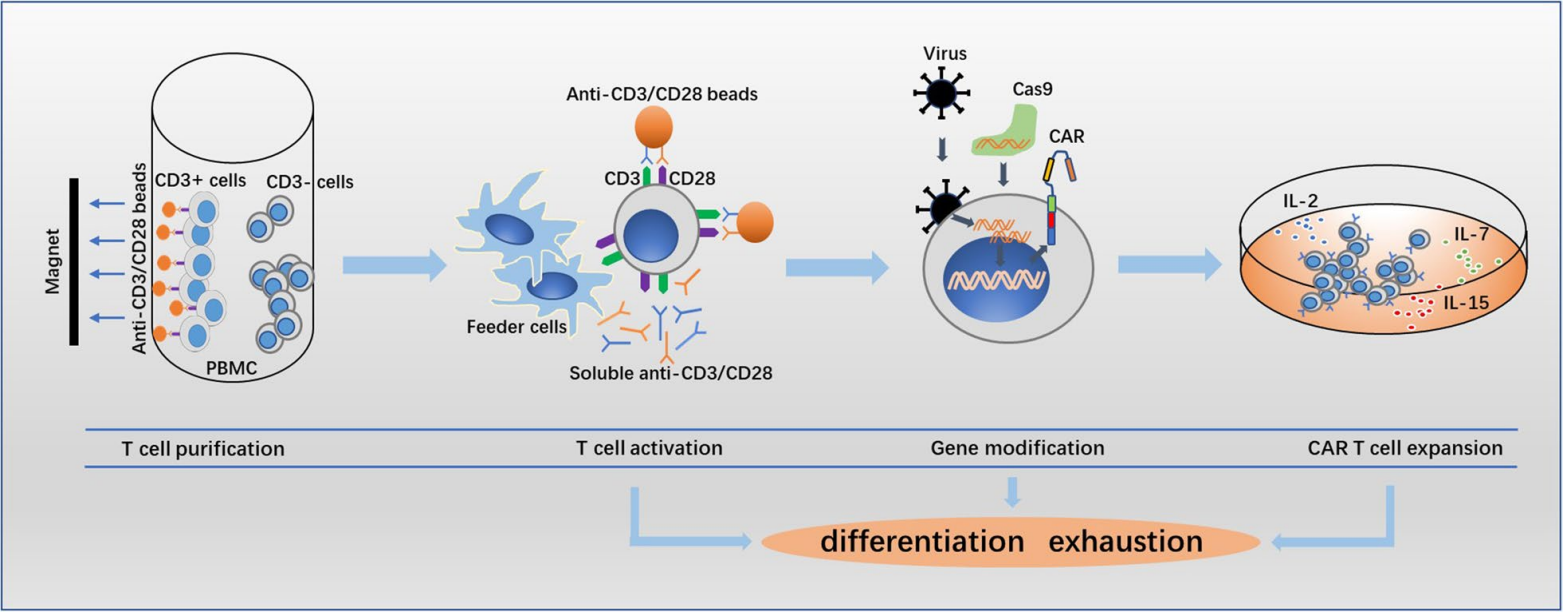
The methods applied in the process of chimeric antigen receptor T cells culture influence cell differentiation and exhaustion state of the final product. In vitro culture process of CAR T cells mainly includes T cell collection, purification, activation, gene transduction and cell expansion. Enrichment of T cells by magnetic adsorption of CD3/CD28 antibody coated beads is commonly used but the purity cannot be guaranteed. Different methods for T cell activation leading to different activation strength or activity of pathways, and the viral or non-viral gene editing resulting in random or site-specific gene integration, ultimately influence the state of cell differentiation and exhaustion. Reducing the duration of ex vivo culture yields less differentiated CAR T cells, and addition of cytokines such as IL-7, IL-15 and IL-21 increases the frequencies of memory stem CAR T cells

Generally, in vitro culture process of CAR T cells includes T cell collection, purification, activation, gene transduction and cell expansion. The methods applied in each step may influence the cell composition or state of the final product (Fig. 1).

### T cells collection and purification

Variability in CAR T cells expansion is in part due to the contamination of the starting peripheral blood mononuclear cells (PBMC) concentrated with monocytes. Among patients whose CAR T cells expanded poorly, manufacturing incorporating a monocyte depleting plastic adherence step could yield an adequate dose of CAR T cells for clinical use [59]. A density-based negative selection for T cells enrichment using antibody cocktail presents the advantage of leaving T cells free of antibodies [60], thereby reducing the probability of excessive or inadequate cells activation. The most commonly used method for enrichment of T cells is magnetic adsorption using CD3/CD28 antibody coated beads, but the purity of T cells can’t be guaranteed. The CAR gene may be transduced into leukemic cells during T cell manufacturing, and its product bound in cis to the CD19 epitope on the surface of leukemic cells, masking it from recognition by and conferring resistance to CD19-targeted CAR T cells [61]. Therefore, the existence of CAR-tumor cells needs to be taken into consideration when relapse occurs, and the methods for T cells purification need further exploration.

### T cells activation

Anti-CD3/CD28 beads stimulation provided a suitable activation for clinical use of genetic modification of T cells [62], as well as the application of soluble anti-CD3/CD28 antibody or anti-CD3/CD28 coated plate. Of note, in addition to anti-CD3 for T cell activation, anti-CD28 is needed to avoid T cell anergy. Compared to soluble anti-CD3, anti-CD3/CD28 beads stimulated greater CD4 + T cells growth, but both stimulated similar CD8 expansion [63]. The conjugation of anti-CD137 antibodies to conventional anti-CD3/CD28 beads results in a significant increase in the expansion capacity for CD8 cytotoxic T cells [64]. Besides, irradiated feeder cells from PBMC in the presence of anti-CD3 antibody have been shown to be a high effective approach for expansion of CD8 + T cells with a high ratio of central memory-like T cells [65].

### Gene transduction

The most common and effective methods for gene transduction in CAR T cells production is using viral vectors carrying CAR transgene, including γ-retrovirus and lentivirus. These vectors are widely used for T cells modification and are successful in clinical trials due to the stable and durable expression of target genes by integration into the genome of host cells. However, the random integration of viral vectors may result in variegated transgene expression which ultimately influence the in vivo persistence of CAR T cells. The non-viral gene editing, especially the CRISPR/Cas9-mediated site-specific gene integration, results in uniform CAR expression, which averts tonic CAR signaling and establishes effective internalization and re-expression of the CAR following single or repeated exposure to antigen, delaying effector T cells differentiation and exhaustion [66].

### CAR T cells expansion

In order to obtain sufficient amount of CAR T cells, a proper duration for T cell expansion ex vivo is necessary. Most protocols for T cells engineering routinely expand T cells for 9–14 days, but there is a trend of shortening recently. The potential for engraftment and persistence of CAR T cells are related to the state of T cells differentiation, and reducing the duration of ex vivo culture could limit cells differentiation and enhance the efficacy of CAR T cells therapy [67].

IL-2 is necessary for T cell culture, but it may drive T cell differentiation. Low concentration or short-term use of IL-2 favors generation of early memory T cells over effector phenotypes during CAR T cells expansion, thereby enhancing therapeutic efficacy and saving production cost [68, 69]. Addition of soluble IL-7, IL-15 and/or IL-21 in the process of cell culture can reduce the CAR T cells terminal differentiation and increase the frequency of memory stem cells, yielding improved in vivo persistence [70–72].

## Activation signaling

### CAR signaling

T-cell activation is a necessary step in the production of CAR T cells, and the robust cytotoxicity of CAR T cells depend on the antigen recognition by chimeric antigen receptors and subsequent activation signaling. However, excessive activation drives T cells differentiation and exhaustion [73], which rationalizes the notion that appropriate activation is essential to optimize therapeutic potency and durability of CAR T cells. Modulation of multiple links in the activation signal pathway can affect the state of CAR T cells differentiation and exhaustion. With CARs encoding a single ITAM, CAR T cells show less differentiation and exhaustion [23]. Inhibition of PI3K preserves a less differentiated state of CAR T cells without affecting cell expansion [74, 75]. MAP Kinase inhibition protects tumor-infiltrating CD8 T cells from death driven by chronic TCR stimulation [76]. Akt inhibitor treated CAR T cells present an early memory phenotype and superior antitumor efficacy owing to the intranuclear localization of T cell memory associated transcriptional regulator FOXO1 [77, 78]. Different from unmodified T cells, CAR-modified T cells with sustained activation signaling induced by spontaneous aggregation of CAR molecules lead to cell differentiation and exhaustion during ex vivo expansion, limiting their therapeutic efficacy and in vivo persistence [16, 74]. Thus, in addition to direct regulating activation signaling, it might be useful to modulate the density of CAR molecules on the cell surface in order to prevent the aggregation of CARs induced sustained cell activation. This is consistent with the observations that the level of CAR surface expression correlates with the in vivo persistence of CAR T cells, and the density of CAR can be controlled by modifying the promoters.

### TCR signaling

The initiation of T cell antigen receptor (TCR) signaling is a key step that result in T cell activation and the orchestration of an adaptive immune response. Theoretically, CAR T cells retain the structure and function of TCR which can still recognize specific antigens and transduce activation signals. The dual stimulation of CAR and TCR may lead to loss of CD8 CAR T cells efficacy owing to excessive activation induced cell exhaustion and apoptosis [79]. Allogeneic CD19- specific CAR T cells- mediated anti-lymphoma activity without causing a significant increase in the incidence of graft-versus-host disease (GVHD) also suggests that both the TCR and CAR are engaged to accelerate T cells exhaustion [80]. CAR T cells with TCR knock-out exhibit high anti-leukemic effects and long-term persistence in the absence of alloreactivity [81]. Directing a CD19-targeted CAR to the alpha constant locus of TCR which undergoes structural disruption not only results in uniform CAR expression, but also enhances CAR T cells therapeutic potency with delayed effector T cells differentiation and exhaustion [66].

## Epigenetic regulation

Epigenetic regulation including DNA methylation, histone modification and chromatin remodeling plays a very important role in T cells differentiation by modulating the transcription of differentiation related genes on and off [82]. The loss of methylcytosine dioxygenase TET2 promotes CD8 T cell memory differentiation [83]. A single CAR T cell with disrupted TET2 gene by CAR transgene insertion expanded continuously so as to induced a durable complete remission in a patient with chronic lymphocytic leukemia (CLL), and further research demonstrated that TET2-disrupted CAR T cells exhibited superior expansion with a central memory phenotype [84]. DNA methyltransferase DNMT3a which localizes to the TCF7 promoter is a crucial regulator of CD8 early effector cell differentiation [85]. The demethylation drug decitabine treated CD123 CAR T cells are enriched in genes associated with naïve and early memory T cells with decreased expression of DMNT3a and DNMT1 [86]. The role of epigenetic regulation in T cell exhaustion also should not be neglected. Exhaustion related hypermethylation which was found in tumor infiltrating T cells can be reversed by decitabine [87]. Therefore, epigenetic modification can be utilized to develop new type of CAR T cells with superior performance in clinical application.

## CD4 and CD8 CAR T cells

It is generally believed that the efficacy of adoptive cell therapy is most often attributed to CD8 T cells [88], and infusion of CD8 CAR T cells alone was sufficient for long-term B-cell eradication [89, 90]. CD4 T cells are known for their helper function and to intrinsically evoke cytolytic activities by enhancing CD8 T cells activity through cytokine production [91]. However, CD4 CAR T cells demonstrated comparable effectiveness in directly killing target tumor cells both in vitro and in vivo [79, 92, 93]. In terms of characteristics of killing dynamics, CD4 CAR T cells show initial slower granzyme B secretion and tumor killing, but are less prone to AICD and exhaustion compared to CD8 counterparts, which confers CD4 CAR T cells a relative better persistence following antigen exposure [93–96]. In most of reported clinical trials, patients have received CAR T cells products comprising random compositions of CD4 and CD8 cells, but the variation may have influenced the efficacy. Defining the 1:1 ratio of CD4:CD8 CAR T cells confers superior antitumor reactivity in vivo, indicating the synergistic antitumor effects of the two subsets [97, 98].

## Tumor environment

**Fig. 2:**
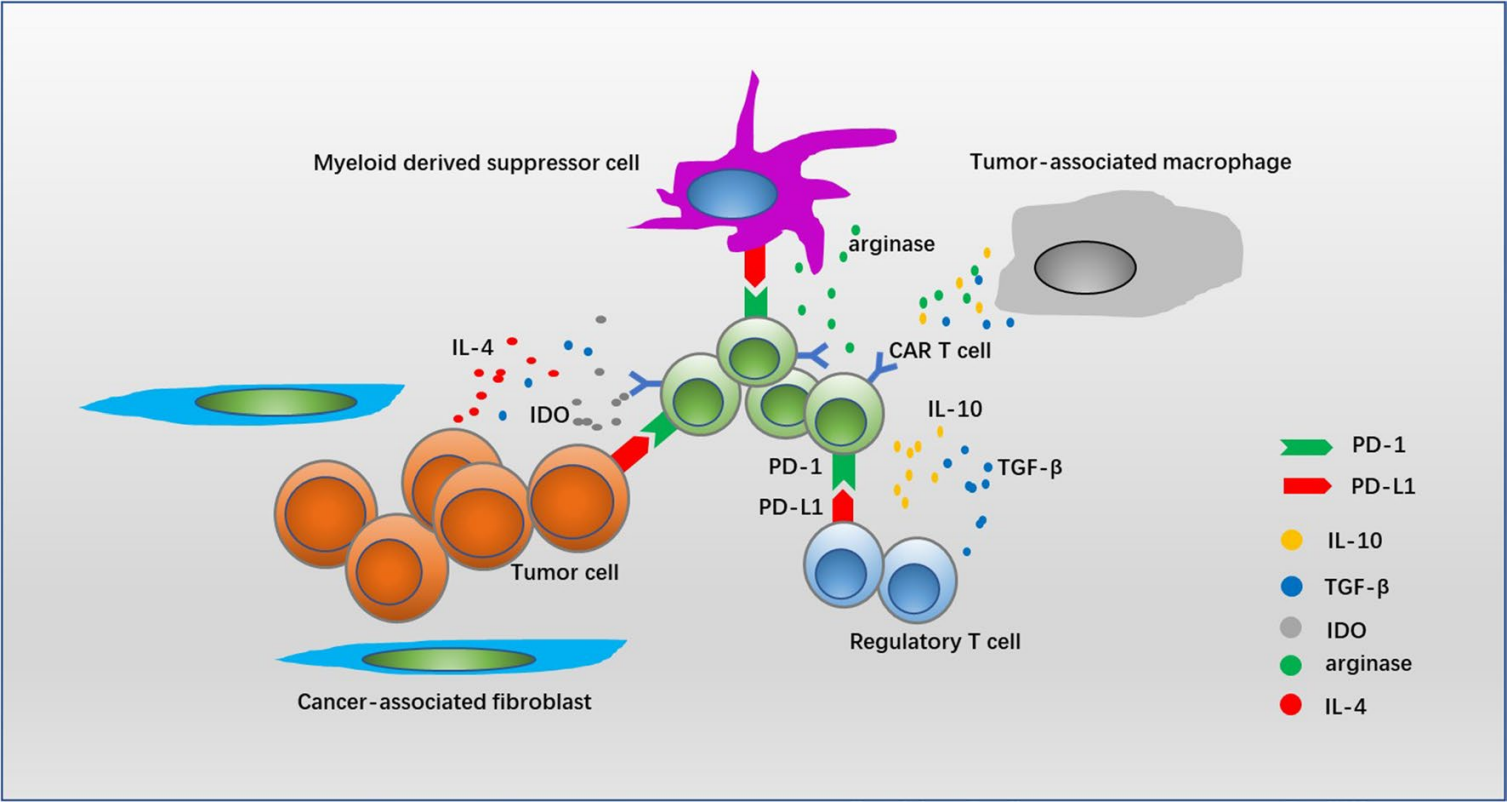
Tumor cells which orchestrate an immune inhibitory microenvironment compromise the activity of CAR T cells. Tumor cells, immunosuppressive cells (e.g., myeloid derived suppressor cells and regulatory T cells) and tumor-associated stroma cells inhibit the activity of CAR T cells by expression of inhibitory molecules (e.g., PDL1) and release of inhibitory cytokines such as IL-10, indoleamine 2,3-dioxygenase (IDO), transforming growth factor-β (TGF-β), arginase and IL-4. In this immunosuppressive microenvironment, tumor infiltrating CAR T cells rapidly lose their cytolytic and cytokine secretion capacity, and tend to be exhaustion. Approaches targeting suppressive factors can improve the efficacy of CAR T cells

Some certain types of cancer cells protect themselves from the attack of immune system by expression of immune inhibitory ligands such as PD-L1 [99, 100], and release of the immune inhibitory cytokines such as indoleamine 2,3-dioxygenase (IDO) [101], arginase [102], TGF-β [103] and IL-4 [104], which impair the activity of CAR T cells through different mechanisms. Moreover, tumor orchestrates an immune inhibitory microenvironment, containing regulatory immune cells such as regulatory T cells (Tregs) and myeloid derived suppressor cells (MDSCs), which also compromise the effector function of CAR T cells through inhibitory ligands (e.g. PD-L1) and/or cytokines (e.g., IL-10, TGF-β and arginase) [105, 106]. Tumor infiltrating CAR T cells rapidly lose their cytolytic and cytokine secretion capacity, and tend to be exhaustion [107]. After “rest” away from the tumor microenvironment, the expression levels of inhibitory receptors (e.g., PD1, TIM3 and LAG3) decreased dramatically [108]. These results suggest that the immunosuppressive tumor microenvironment significantly impacts the effective antitumor responses generated by CAR T cells (Fig. 2). It has been reported that refractory diffuse large B-cell lymphoma with high expression of PD-L1 failed to respond to CD19-targeted CAR T cells therapy, but following PD-1 blockade, CAR T cells expanded with clinically significant antitumor response [109]. IDO inhibits CAR T cells activity through the action of tryptophan metabolites, while fludarabine and cyclophosphamide which are frequently administered before CD19-targeted CAR T cells infusion downregulate the expression of IDO in B cell malignancies and thereby improve CAR T cells activity [101]. In order to eliminate the suppressive effect of TGF-β in tumor environment, CAR T cells expressing the dominant-negative TGF-βRII demonstrate resistance to exhaustion and long-term persistence in prostate cancer mouse models [103]. Fusing the IL-4 receptor exodomain to IL-7 receptor endodomain inverts the inhibitory effects of tumor-derived IL-4 and instead promotes CAR T cells proliferation [104]. Pharmacological administration such as fludarabine and/or cyclophosphamide targeting Tregs, doxorubicin or all-trans retinoic acid targeting MDSCs effectively impairs regulatory immune cell-induced immunosuppression, therefore fostering the efficacy of CAR T cells therapy [110–112]. Besides, tumor-associated stroma, represented by cancer-associated fibroblasts (CAFs), can also contribute to form a highly pro-tumorigenic immunosuppressive microenvironment that mediates immunotherapeutic resistance, while combining the stroma and tumor-targeted CAR T cells significantly enhanced overall antitumor activity [113].

## CAR T cells differentiation and exhaustion

**Fig. 3:**
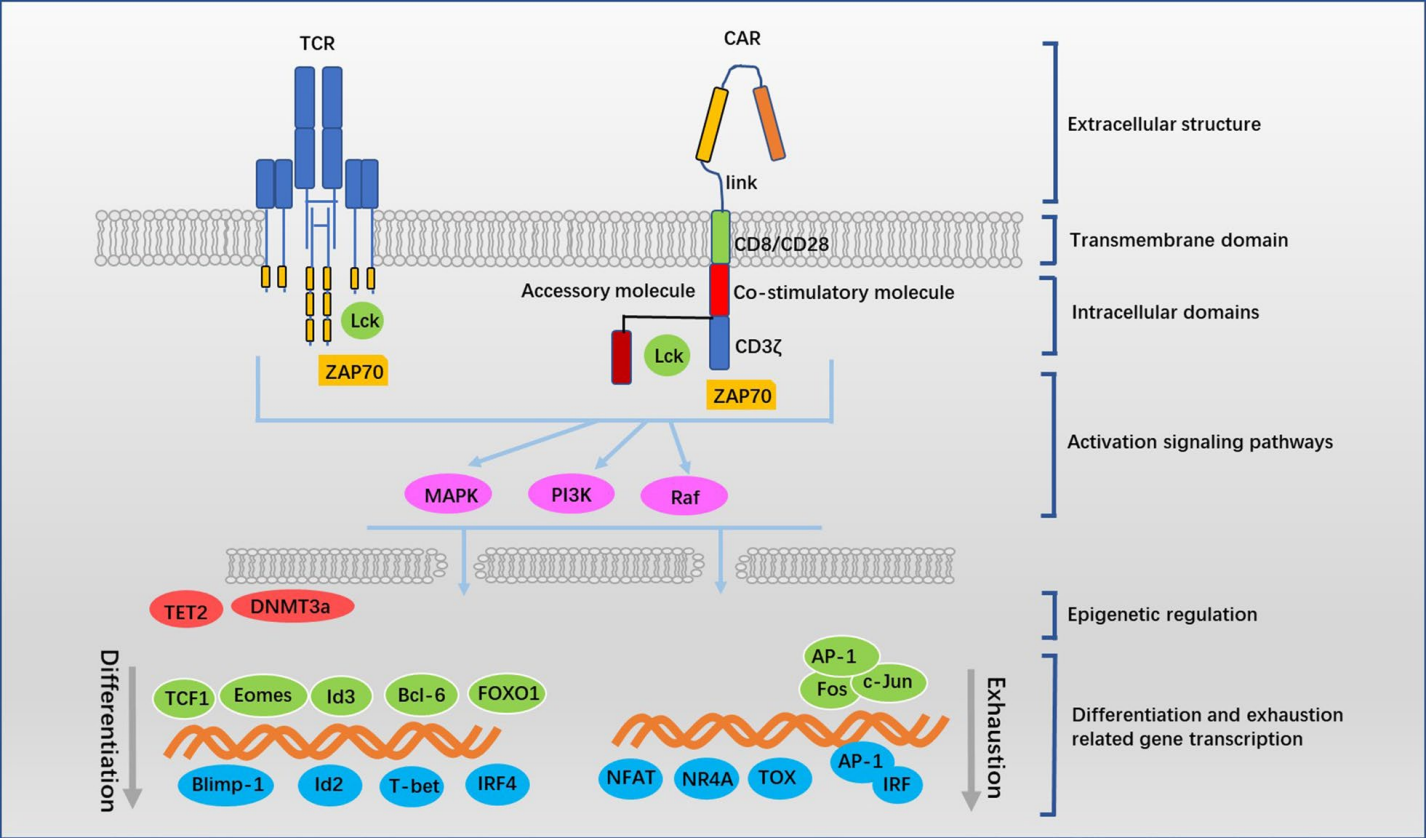
The CAR structures, activation signaling and epigenetic regulators independently or collaboratively participate in CAR T cells differentiation and/or exhaustion. The differences in CAR structures (Extracellular structures, transmembrane and intracellular domains, the accessory structures), CAR signaling and TCR engagement lead to differences in activation strength and duration, as well the activation pathways, which further influence the expression or activities of differentiation and/or exhaustion-associated transcription factors and epigenetic regulators, and ultimately CAR T cells manifest as various degrees of differentiation and/or exhaustion during ex vivo expansion or following in vivo antigen exposure. Thereby, cell differentiation and exhaustion which may lies at the central link in determining CAR T cells in vivo persistence, can be modulated by intervention of the above influence factors

From the above, CAR structure, activation signaling, epigenetic regulations, methods for in vitro culture and tumor environment, all of which independently or collaboratively influence the state of CAR T cells differentiation and/or exhaustion in various degrees through different mechanisms (Fig. 3). Thus, differentiation and exhaustion may be the central part and share a certain range of common pathways in impacting the CAR T cells in vivo persistence.

### The stage of CAR T cells differentiation

According to the stage of cell differentiation, T cell can be categorized as Naive T cell (T_N_), stem cell memory T cell (T_SCM_), central memory T cell (T_CM_), memory effector T cell (T_EM_), effector T cell (T_EF_). It is well defined that the therapeutic efficacy and in vivo persistence of CAR T cells significantly correlate with their differentiation stage. CAR T cells products rich in T_N_,T_CM_ and T_SCM_ exhibit superior antitumor responses and long-term persistence in vivo [98, 114, 115]. Both of CD8 and CD4 CAR T cells derived from TN and T_CM_ are more effective than those from T_EM_ [98]. Transcriptomic profiling revealed that CAR T cells from complete-responding patients with CLL were enriched in memory-related genes, whereas CAR T cells from non-responders upregulated programs involving effector differentiation, and sustained remission was associated with an elevated frequency of memory-like T cells before CAR T cells generation [116].

Most of current approaches for maintaining durable memory CAR T cells in vivo are improving the capacity of memory formation in the stage of manufacture. These approaches including design refinement of CAR structure, control of activation signaling, epigenetic regulations and optimization of in vitro culture, ultimately regulate the expression or activities of T cell differentiation-associated transcription factors (TFs). Numerous TFs have been identified as critical regulators of conventional CD8 T cell differentiation, including memory-associated TFs (e.g., TCF-1, Eomes, Id3, Bcl-6 and FOXO1) and effector-associated TFs (e.g., T-bet, Blimp-1, Id2, and IRF4) [117, 118]. Considering that CAR T cells may share the similar differentiation-associated transcriptional regulations as conventional T cell, gene modifications or small molecule drugs targeting these TFs may be promising for CAR T cells memory maintenance.

Upon antigen exposure, naive T cells are activated and differentiate into multiple types of effector T cells, and the differentiation is accompanied by robust proliferation, transcriptional, epigenetic and metabolic reprogramming [117, 119]. Following the peak of effector expansion, the resolution of inflammation and the clearance of antigen, most activated T cells die, but a subset persists and transits into the memory T cells pool which maintains the ability to rapidly reactivate effector functions upon re-stimulation [120]. CAR T cells may undergo the similar process of differentiation following antigen recognition after therapeutic infusion. However, the strategies of in vivo intervention for CAR T cells memory maintenance still need further exploration.

### CAR T cells exhaustion

CAR T cells present less effective always because of exhaustion which occurs both in vivo and in vitro. During in vitro expansion, spontaneous CAR molecule aggregation-induced sustained tonic signaling lead to CAR T cells exhaustion [16]. After therapeutic infusion, upon repeated antigen stimulation, part of CAR T cells enter into an exhausted state which is defined by poor effector function, sustained expression of inhibitory receptors (e.g., PD1, TIM3 and LAG3) and widespread transcriptional and epigenetic alterations distinct from that of functional effector or memory T cells [73, 120, 121].

At the level of transcription, AP-1 related bZIP-IRF families have been identified as major modulators that drive exhaustion-associated gene expression. Exhaustion-associated dysfunction resulted from increased levels of AP-1/IRF complexes leads to a functional deficiency in activating AP-1 Fos/Jun heterodimers, whereas c-Jun overexpression prevents phenotypic and functional hallmarks of exhaustion and improves antitumor efficacy [60]. NFAT/NR4A axis which controls the expression of multiple inhibitory receptors plays a crucial role in T cell exhaustion, and CAR T cells lacking all three NR4A transcription factors results in tumor regression and prolonged survival in tumor-bearing mice [122, 123]. Transcription factor TOX cooperates with NR4A to regulate T cells exhaustion [124, 125], and disruption of TOX activity could be a promising strategy for enhancing therapeutic potency of CAR T cells.

Inhibitory receptors such as PD1, TIM3 and LAG3 are recognized as the markers of T cells exhaustion, although they also increase significantly in response to cell activation [126]. Tumor cells or tissues up-regulate the inhibitory ligands (e.g., PDL1) upon immune attack, while CAR T cells up-regulate the inhibitory receptors (e.g., PD1) in response to antigen exposure, and the PDL1/PD1 pathway significantly inhibits CAR T cells activity. Thus, intervention of PDL1/PD1 pathway emerges as a promising approach for reinvigorating CAR T cells in certain clinical application. Strategies for intervention of PDL1/PD1 axis developed recently involving CAR T cells modified to secrete PD1 blocking scFv [127], disruption of PD1 by gene editing [128, 129], and the application of monoclonal antibodies targeting PDL1 or PD1. Of note, compared to 4-1BB/CAR T cells, CD28/CAR T cells are more susceptible to exhaustion that PD-1/PD-L1 blockade confers a priority [130, 131]. And even, downregulation of three inhibitory receptors (PD1, TIM3 and LAG3) simultaneously on CAR T cells show increased tumor infiltration and durable tumor control [132].

The exhaustion of CAR T cells limits their efficacy and durability in vivo, but in certain application, exhaustion may present a positive effect. Due to the exhausting alloreactivity caused by cumulative TCR and CAR signaling, allogeneic CAR T cells demonstrated reduced risk of GVHD in mice model [80, 133].

## Conclusions

CD19-targeted CAR T cells therapy has shown remarkable efficacy in treatment of B cell malignancies, which opens up a new horizon in the field of cancer therapy. However, the ability of cancer cells to modify themselves and adapt the new survival stress, the complicated regulatory mechanisms of CAR T cells and tumor microenvironment, as well as the adverse effects such as cytokines release syndrome and on-target off-tumor toxicity [134], make adoptive T cells immunotherapy still face many challenges. Scientists are making great efforts to reveal the mechanisms and propose reliable solutions to impediments in CAR T cells therapy. In this rapidly developing field, CAR T cells with superior performance will be applied in more and more cancers.

## Data Availability

The material supporting the conclusion of this review has been included within the article.

## Abbreviations

CAR: Chimeric antigen receptor
scFv: Single-chain variable fragment
B-ALL: B acute lymphoblastic leukemia
CLL: Chronic lymphocytic leukemia
CR: Complete remission
AICD: Activation-induced cell death
SRSF3: Serine and arginine rich splicing factor 3
PAX5: Paired box 5
EBF1: Early B cell factor 1
ITAMs: Immunoreceptor tyrosine-based activation motifs
ICOS: Inducible co-stimulator
CD28-YMFM: Changing a single amino acid residue asparagine to phenylalanine in CD28 domain
MyD88: Myeloid differentiation primary response gene 88
Bcl-XL: BCL2L1, BCL2 like 1
OX40: TNFRSF4, tumor necrosis factor receptor superfamily member 4
TLR2: Toll-like receptor 2
Tregs: Regulatory T cells
MDSCs: Myeloid derived suppressor cells
CAFs: Cancer-associated fibroblasts
TCR: T cell receptor
PBMC: Peripheral blood mononuclear cells
TN: Naive T cell
TSCM: Stem cell memory T cell
TCM: Central memory T cell
TEM: Memory effector T cell
TEF: Effector T cell
GVHD: Graft-versus-host disease
CRISPR/Cas9: Clustered regularly interspaced short palindromic repeats/CRISPR-associated 9
MAPK: Mitogen-activated protein kinase
JAK/STAT: Janus kinase and signal transducer and activator of transcription
IRF7/IFN-β: Interferon regulatory factor 7 and interferon-β
PI3K: Phosphatidylinositol 3 kinase
TCF-1: Transcription factor-1
TET2: Tet methylcytosine dioxygenase 2
DNMT3a: DNA methyltransferase 3 alpha
Eomes: Eomesodermin
Id2/3: Inhibitor of DNA binding 2/3
Bcl-6: B cell leukemia/lymphoma 6
FoxO1: Forkhead box O1
T-bet: TBX21,T-box transcription factor 21
Blimp-1: PRDM1, PR/SET domain 1
IRF4: Interferon regulatory factor 4
NFAT: Nuclear factor of activated T
NR4A: Nuclear receptor subfamily 4 group A
TOX: Thymocyte selection associated high mobility group box
IL-2: Interleukin-2
IL-4: Interleukin-4
IL-7: Interleukin-7
IL-15: Interleukin-15
IL-21: Interleukin-21
IL-12: Interleukin-12
IL-23: Interleukin-23
CCL12/19: C–C motif chemokine ligand 12/19
IDO: Indoleamine 2,3-dioxygenase
TGF-β: Transforming growth factor-β
PD-1: Programmed death 1
TIM3: T cell immunoglobulin domain and mucin domain 3
LAG3: Lymphocyte activating 3
